# 

**Published:** 1859-05

**Authors:** 


### LIST OF COLLABORATORS.

S. G. Armor, M.D., late Professor of Pathology and Clinical Medicine, Missouri Med. College.
John L. Atlee, M.D., late President Pennsylvania State Medical Society.
Walter F. Atlee, M.D., Philadelphia.
Robert G. Barclay, M.D., Jerusalem, Syria.
John Bell, M.D., Philadelphia, formerly Professor of Medicine, Medical College of Ohio.
T. S. Bell, M.D., Professor of Medicine, University of Louisville.
S. M. Bemiss, M.D., Professor of Clinical Medicine, University of Louisville.
L. P. Blackburn, M.D., New Orleans, La.
George C. Blackie, M.D., Professor of Botany, University of Nashville.
G. C. Blackman, M.D., Professor of Surgery, Medical College of Ohio.
J. L. Bobbs, M.D., formerly Professor of Anatomy, Indianapolis.
W. M. Boling, M.D., Montgomery, Ala., formerly Professor of Midwifery, Transylvania University, Lexington, Ky.
W. S. Bowen, M.D., New York.
N. Bozeman, M.D., Montgomery, Ala.
J. T. Bradford, M.D., Augusta, Ky.
John H. Brinton, M.D., Lecturer on Operative Surgery, Philadelphia.
W. S. Chipley, M.D., Professor of Medicine, Transylvania University.
A. Clapp, M.D., New Albany, Ia.
D. B. Cliffe, M.D., Franklin, Tenn.
J. Da Costa, M.D., Lecturer on the Practice of Medicine at the Philad. Med. Association.
Samuel H. Dickson, M.D., Professor of Medicine, Jefferson Medical College.
M. Dupierris, M.D., Havana, Cuba.
Richard J. Dunglison, M.D., Philadelphia.
W. F. Edgar, M.D., United States Army.
S. C. Farrar, M.D., Jackson, Miss.
C. S. Fenner, M.D., Memphis, Tenn.
Emil Fischer, M.D., Philadelphia.
Austin Flint, M.D., Professor of Clinical Medicine, Auscultation and Percussion, New Orleans School of Medicine.
Charles Frick, M.D., Baltimore.
W. Y. Gadbury, M.D., Benton, Miss.
O. C. Gibbs, M.D., Chautauque County, New York.

Dr. J. P. HALL, Glasgow, Ky.

WILLIAM A. HAMMOND, M.D., United States Army.

JOHN HARDIN, M.D., Professor of Obstetrics, Kentucky School of Medicine, Louisville.

EDWARD HARTSHORNE, M.D., One of the Surgeons to Wills Hospital for Diseases of the Eye, Philadelphia.

E. B. HASKINS, M.D., Professor of Chemistry, Shelby Medical College, Nashville, Tenn.

C. A. HENTZ, M.D., Marianna, Florida.

ADDINELL HEWSON, M.D., One of the Surgeons to Wills Hospital for Diseases of the Eye, Philadelphia.

SAMUEL L. HOLLINGSWORTH, M.D., late Editor of the Medical Examiner, Philadelphia.

WILSON JEWELL, M.D., Philadelphia, President of the Quarantine and Sanitary Convention.

WM. V. KEATING, M.D., Lecturer on Obstetrics and the Diseases of Women, at the Philadelphia Medical Association.

JOHN G. KERR, M.D., Canton, China.

G. A. KUNKLER, M.D., Madison, Ia.

BEN. LOGAN, M.D., Shreveport, La.

A. LOPEZ, M.D., Mobile, Ala., formerly President of the Alabama State Medical Society.

J. AITKEN MEIGS, M.D., Professor of the Institutes of Medicine in the Philadelphia College of Medicine.

S. WEIR MITCHELL, M.D., Lecturer on Physiology at the Philad. Med. Association.

GEORGE R. MOREHOUSE, M.D., Philadelphia.

B. I. RAPHAEL, M.D., New York.

J. C. REEVE, M.D., Dayton, Ohio.

L. C. RIVES, M.D., formerly Professor of Midwifery, Medical College of Ohio.

J. LAWRENCE SMITH, M.D., Professor of Chemistry, University of Louisville.

W. C. SNEED, M.D., Frankfort, Ky., late President Kentucky State Medical Society.

C. H. SPILMAN, M.D., Harrodsburg, Ky., late President of Kentucky State Medical Society.

GEO. SUTTON, M.D., Aurora, Ia.

W. L. SUTTON, M.D., Georgetown, Ky., formerly President Kentucky State Medical Society.

HORATIO R. STORER, M.D., formerly one of the Physicians to the Boston Lying-in Hospital, Boston, Mass.

S. THOMPSON, M.D., Albion, Ill., late President of the Illinois State Medical Society.

E. WILLIAMS, M.D., Cincinnati, Ohio.

### Bi-Monthly Bibliographical Record.

AMERICAN.

Five Essays. By John Kearsley Mitchell, M.D., etc. Edited by S. Weir Mitchell, M.D., etc. 12mo., pp. 372. Philadelphia: J. B. Lippincott & Co., 1859.

Trials of a Public Benefactor, as illustrated with Discovery of Etherization. By Nathan P. Rice, M.D. 12mo., pp. 460. New York: Pudney & Russell, 1859. (From the publishers.)

Transactions of the Medical Society of the State of Pennsylvania, at its Annual Session, held in Lancaster, May, 1858. New series, part iii. 8vo., pp. 127. Philadelphia, 1858.

Report of the State of the New York Hospital and Bloomingsdale Asylum, for the year 1858. 8vo., pp. 38. New York, 1859. (From Dr. Baylies.)

Report on the Functions of the Cerebellum. By E. Andrews, M.D. Reprint. 8vo., pp. 19. Philadelphia, 1858. (From the author.)

A Paper on the Management of the Shoulders in Examinations of the Chest; including a new Physical Sign. Read before the New York Academy of Medicine, by John W. Corson, M.D., etc. Reprint. 8vo., pp. 32. Ballière: New York, 1859. (From the author.)

Report of the Pennsylvania Hospital for the Insane, for the year 1858. By Thomas S. Kirkbride, M.D., etc. 8vo., pp. 62. Philadelphia, 1859. (From the author.)

Some Account of the Recent Experiments made in Connection with the Case of M. Groux. By J. B. Upham, M.D. Reprint. 8vo., pp. 10. Boston, 1859. (From the author.)

Report of the Trustees and Superintendent of the State Lunatic Asylum of Pennsylvania, for 1858. Reprint. Harrisburg, 1859. (From Dr. Dock.)

FOREIGN.

On Poisons in Relation to Medical Jurisprudence and Medicine. By Alfred S. Taylor, M.D., F.R.S., etc. Second American from second revised London edition. 8vo., pp. 755. Philadelphia: Blanchard & Lea, 1859. (From the publishers.)

A Practical Treatise on the Diseases of Infancy and Childhood. By T. H. Tanner, M.D., F.L.S., etc. 12mo., pp. 464. Philadelphia: Lindsay & Blakiston, 1859. (From the publishers.)

Third Report of the Clinical Hospital, Manchester. By James Whitehead, M.D. 8vo., pp. 117. London: Churchill, 1859. (From the author.)

The Irritable Bladder: its Causes and Curative Treatment. By Frederick James Gant, M.R.C.S., etc. Post 8vo., pp. 136. London: Churchill, 1859.

Méthodes Nouvelles de Traitement des Maladies Articulaires. Exposition et démonstration faites à Paris en 1858. Par le Professeur A. Bonnet, de Lyon. 8vo. Paris: Bossange et Fils, 1859.

Engravings of the Ganglions and Nerves of the Uterus and Heart; for the Use of Students in Anatomy and Physiology. By Robert Lee, M.D., etc. Royal 4to. London: Churchill, 1859.

Agenda des Médecins et Chirurgiens, pour 1859. Par le Docteur Cazenave; le Professeur Trousseau; le Docteur O. Reveil, et M. Constantin James. 24mo. Paris: Bossange et Fils, 1859.

A Manual of Dental Surgery. By John Tomes, F.R.S., etc. Fcap. 8vo. London: Churchill, 1859.

Hemorrhoids and Prolapsus of the Rectum; their Treatment by the Application of Nitric Acid. 8vo. By Henry Smith, F.R.C.S., etc. London: Churchill, 1859.

On the Hygienic Management of Infants and Children. By S. Herbert Boker, M.D., etc. 8vo. London: Churchill, 1859.

Trachéotomie (de la) dans le Croup. Par M. Bouvier, Médecin de l'Hôpital des Enfants, etc. Second discours prononcé à l'Académie Impériale de Médecine (séance du 18 Janvier, 1859.) 8vo. Bossange et Fils: Paris, 1859.

Mémoir sur la Désarticulation Totale de la Mâchoire Inférieure, lu à l'Académie des Sciences, le 10 Août, 1857. Par M. le Docteur J. G. Maisonneuve, Chirurgien de l'Hôpital de la Pitié. 4to. Paris: Bossange et Fils.

General Debility and Defective Nutrition; their Causes, Consequences, and Treatment. By Alfred Smee, F.R.S., etc. 8vo. London: Churchill, 1859.

On the Operations for Strangulated Hernia. By J. H. James, F.R.C.S. 8vo. London: Churchill, 1859.

Ricord's Lectures on Chancre. Edited and translated from the French, by C. F. Maudner, F.R.C.S. 8vo. Churchill: London, 1859.

Neuralgia and Paraplegia from long-continued Use of Arsenic; and other Contributions to Vol. IX. of Transactions of Pathological Society of London. By George D. Gibb, M.D., etc. Reprint. 1858. (From the author.)

